# Temporal Modulation of Type I Interferon and NF-κB Signaling by Baicalin Suppresses Infectious Bronchitis Virus Replication and Inflammatory Response

**DOI:** 10.3390/ani15233396

**Published:** 2025-11-25

**Authors:** Jiongjie He, Ling Wang, Yong Zhang, Shengyi Wang

**Affiliations:** 1 College of Veterinary Medicine, Gansu Agricultural University, Lanzhou 730070, China; ajie7298@163.com; 2 Key Laboratory of New Animal Drug Project of Gansu Province, Key Laboratory of Veterinary Pharmaceutical Development, Ministry of Agriculture and Rural Affairs, Lanzhou Institute of Husbandry and Pharmaceutical Sciences of Chinese Academy of Agriculture Sciences, Lanzhou 730050, China; wangling02@caas.cn

**Keywords:** baicalin, infectious bronchitis virus, NF-κB pathway, type I interferon, IRF3

## Abstract

Background: Infectious bronchitis virus (IBV) poses a significant threat to poultry health, and exploring effective antiviral agents is of considerable importance. This study aimed to investigate the antiviral activity of baicalin against IBV and its underlying immunomodulatory mechanisms. Methods: The antiviral effect of baicalin was evaluated in chicken embryo kidney cells using various treatment schedules. Viral replication was assessed through nucleocapsid protein expression and viral titers. Mechanistic studies involved analysis of key signaling pathways, IRF3 knockdown experiments, and proteasomal degradation assays. Results: Baicalin (20 µg/mL) significantly inhibited IBV replication, with optimal efficacy observed when administered at 2 h post-infection. The compound demonstrated time-dependent regulation of antiviral and inflammatory pathways, enhancing the MDA5-MAVS-STAT1 axis and IFN-β signaling at late infection stage while showing biphasic modulation of NF-κB pathway. IRF3 was identified as essential for both anti-inflammatory and antiviral effects. Baicalin enhanced IκBα stability by inhibiting its proteasomal degradation. Conclusions: Baicalin suppresses IBV replication through coordinated enhancement of type I interferon response and temporal regulation of NF-κB pathway, with IRF3 serving as a key regulatory molecule. These findings provide mechanistic support for baicalin’s potential as an anti-IBV therapeutic agent.

## Simple Summary

Infectious bronchitis virus is a major threat to poultry farming, causing severe economic losses, and current vaccines have limitations. This study investigated whether baicalin, a natural compound from a medicinal plant, could fight this virus. We found that baicalin effectively reduced virus replication in chicken kidney cells, especially when given after infection. It worked by boosting the cells’ own early antiviral defenses, specifically the type I interferon pathway. At the same time, it carefully controlled the inflammatory response, preventing excessive harm that can be caused by the immune system itself. A key protein, IRF3, was identified as crucial for both the anti-inflammatory and antiviral effects. Our results suggest that baicalin could be developed as a promising natural treatment to help control infectious bronchitis in poultry, potentially reducing the reliance on antibiotics and improving animal health and food production.

## 1. Introduction

Infectious bronchitis virus (IBV), a coronavirus that poses a significant threat to the global poultry industry, remains difficult to control due to the co-circulation of multiple serotypes and genotypes. This diversity stems from its highly variable genome and the frequently weak cross-protective immunity among different strains [1]. In addition to characteristic respiratory signs and high mortality, IBV infection can affect multiple systems—including the renal and digestive tracts—leading to persistent infection and substantial economic losses [2]. In recent years, the continuous evolution and global dissemination of genotypes such as QX-like (GI-19) have not only increased pressure on the poultry sector but also raised concerns regarding their potential for cross-species transmission and implications for public health [3,4].

Current IBV research focuses on viral biology, pathogenesis, and vaccine development. Reverse genetics systems, in particular, have provided powerful tools for identifying virulence-associated genes and deciphering patterns of antigenic variation [5]. Although conventional live-attenuated and inactivated vaccines are widely employed, their efficacy is often limited by the ongoing emergence of novel variant strains, thereby limiting broad and durable immune protection [6]. As a result, the development of novel antiviral agents has become an essential research direction, encompassing both small-molecule inhibitors targeting key viral enzymes [7] and biologics capable of inducing broad-spectrum immune responses. Natural products have also attracted considerable interest due to their multi-target mechanisms, low resistance potential, and favorable safety profiles. Natural bioactive compounds—such as flavonoids, polysaccharides, and alkaloids—have demonstrated antiviral activity through diverse mechanisms, including direct inhibition of viral replication, modulation of host immune responses, and mitigation of oxidative damage, suggesting promising green control strategies against IBV [8,9].

Among these natural products, baicalin—a flavonoid derived from Scutellaria baicalensis—exhibits notable antiviral potential. Its mechanism is characterized by polypharmacology: besides directly inhibiting IBV attachment and replication, it alleviates immunopathological damage by modulating signaling pathways such as TLR/NF-κB [10]. Moreover, studies suggest that baicalin has broad-spectrum activity against coronaviruses, including SARS-CoV-2, largely attributable to its inhibition of 3CL protease (Mpro) and PL protease (PLpro), which disrupts viral polyprotein processing [11,12]. However, most existing studies have focused on isolated pathways or individual gene functions. The synergistic relationship between the type I interferon (IFN) and NF-κB inflammatory pathways in the anti-IBV activity of baicalin has not yet been systematically investigated, limiting a holistic mechanistic understanding and full exploitation of its therapeutic potential [13].

Therefore, this study aims to employ an in vitro system to verify the inhibitory effect of baicalin on IBV replication, with a specific focus on systematically elucidating the molecular mechanisms by which baicalin modulates host innate immunity—particularly through the coordinated regulation of type I IFN and NF-κB signaling pathways. This work is expected to provide new insights into the multi-target antiviral mechanisms of baicalin and offer theoretical support for the development of broad-spectrum countermeasures against IBV and other coronaviruses.

## 2. Materials and Methods

### 2.1. Cells, Virus, and Reagents

Primary Chicken Embryonic Kidney (CEK) cells were isolated from 10-day-old specific-pathogen-free (SPF) chicken embryos via trypsin digestion. Cells were cultured in DMEM (Thermo Fisher Scientific, Waltham, MA, USA) supplemented with 10% fetal bovine serum (FBS) (Thermo Fisher Scientific, Waltham, MA, USA), 100 U/mL penicillin and 100 µg/mL streptomycin (Thermo Fisher Scientific, Waltham, MA, USA) at 37 °C in a humidified incubator with 5% CO_2_.

The Infectious Bronchitis Virus (IBV) Beaudette strain was propagated in confluent monolayers of CEK cells. When the cytopathic effect (CPE) reached approximately 80%, the cell culture supernatant was harvested. After three freeze–thaw cycles, the lysate was centrifuged, and the supernatant was aliquoted and stored at −80 °C for subsequent experiments. The viral titer was determined by the CPE-based method and calculated as the 50% tissue culture infectious dose (TCID_50_).

Baicalin standard (purity ≥ 98%) was purchased from Shanghai Yuanye Bio-Technology Co., Ltd. (Shanghai, China). A stock solution of 20 mg/mL was prepared in dimethyl sulfoxide (DMSO) (Sigma-Aldrich, St. Louis, MO, USA), filter-sterilized, aliquoted, and stored protected from light at −20 °C. Ribavirin (Sigma-Aldrich, St. Louis, MO, USA) and the proteasome inhibitor MG132 (Sigma-Aldrich, St. Louis, MO, USA) were also used. Primary antibodies against IBV N protein, MDA5, MAVS, STAT1, p-STAT1, IRF3, p-IRF3, p65, p-p65, IκBα, p-IκBα, IL-1β, IL-6, TNF-α, TLR7, MyD88, and GAPDH were all purchased from Cell Signaling Technology (CST, Danvers, MA, USA). IRF3-specific siRNA and a negative control siRNA were designed and synthesized by Shanghai GenePharma Co., Ltd. (Shanghai, China).

### 2.2. Experimental Groups and Drug Treatment

To evaluate the time-dependent antiviral effect of baicalin, the following experimental groups were established:

Mock group: Uninfected and untreated CEK cells.

IBV group: Cells infected with IBV only (virus control).

Baicalin + 2 h → IBV group (Pre-treatment): Cells were pre-treated with 20 µg/mL baicalin (the optimal concentration of 20 µg/mL for baicalin treatment was established through prior screening, where it yielded the most potent anti-IBV activity while exhibiting no detectable cytotoxicity in CEK cells) [14] for 2 h. The medium was then removed, cells were washed twice with PBS, and subsequently infected with IBV.

IBV + 2 h → Baicalin group (Post-infection treatment): Cells were infected with IBV and adsorbed for 2 h at 37 °C. The viral inoculum was then aspirated, cells were washed twice with PBS, and the medium was replaced with maintenance medium containing 20 µg/mL baicalin.

Baicalin + IBV Co-incubation group: IBV was mixed with an equal volume of baicalin (final concentration 20 µg/mL) and co-incubated at 37 °C for 2 h before infecting the cells.

IBV + 2 h → RT group (Positive control): At 2 h post-infection, the medium was replaced with maintenance medium containing 10 µg/mL Ribavirin.

Unless otherwise specified, the concentration of baicalin used was 20 µg/mL. Samples were collected at 24 or 48 h post-infection for subsequent analysis.

### 2.3. Viral Titer Assay

Viral titers were determined using the CPE-based method. Briefly, collected cell culture supernatants were serially diluted 10-fold in maintenance medium. Each dilution was inoculated onto confluent CEK cell monolayers in a 96-well plate (eight replicates per dilution). Plates were incubated at 37 °C for 72 h and observed daily for CPE. The TCID_50_ was calculated using the Reed–Muench method.

### 2.4. Quantitative Real-Time PCR (qRT-PCR)

Total cellular RNA was extracted using TRIzol reagent. According to the manufacturer’s instructions, RNA was reverse-transcribed into cDNA using a Reverse Transcription Kit. Using cDNA as the template, amplification was performed on a QuantStudio 6 Flex Real-Time PCR System using SYBR Green Premix. The relative mRNA expression levels of target genes were calculated using the 2^−ΔΔCt^ method, normalized to the GAPDH housekeeping gene. Specific primer sequences for detecting the IBV N gene and host immune-related genes were synthesized by Sangon Biotech Co., Ltd. (Shanghai, China) (Table A1).

### 2.5. Indirect Immunofluorescence Assay (IFA)

Cells were cultured and treated in 24-well plates. After treatment, cells were fixed with 4% paraformaldehyde for 15 min and permeabilized with 0.1% Triton X-100 for 10 min. Following blocking with 5% bovine serum albumin (BSA) for 1 h at room temperature, cells were incubated with primary antibodies against IBV N protein or p-p65 overnight at 4 °C. The next day, after washing three times with PBS, cells were incubated with corresponding FITC- or Cy3-conjugated secondary antibodies for 1 h at room temperature in the dark. Cell nuclei were stained with DAPI. Finally, images were observed and captured using a fluorescence microscope.

### 2.6. Western Blot Analysis

Total cellular protein was extracted using RIPA lysis buffer, and protein concentration was determined by the BCA method. Equal amounts of protein samples were separated by SDS-PAGE and transferred onto PVDF membranes. The membranes were blocked with 5% skimmed milk and then incubated with respective primary antibodies (1:1000 dilution) overnight at 4 °C. After washing three times with TBST, membranes were incubated with HRP-conjugated secondary antibodies (1:5000 dilution) for 1 h at room temperature. Protein bands were visualized using an ECL chemiluminescence kit and detected with a chemiluminescence imaging system. GAPDH served as the loading control. Band intensities were quantified using ImageJ software (Version 1.53 t; National Institutes of Health, Bethesda, MD, USA).

### 2.7. Small Interfering RNA (siRNA) Transfection

To knock down IRF3 expression, CEK cells at approximately 70% confluence were transfected with IRF3-specific siRNA or negative control siRNA using Lipofectamine 3000 transfection reagent according to the manufacturer’s instructions (Table A1). After 6 h, the transfection mixture was replaced with complete medium. Cells were cultured for an additional 48 h, and the knockdown efficiency of IRF3 was verified by Western blot before subsequent viral infection and drug treatment experiments.

### 2.8. Statistical Analysis

All experiments were independently repeated at least three times. Data are presented as the mean ± standard deviation (SD). Statistical analysis was performed using GraphPad Prism 8.0 software. One-way analysis of variance (ANOVA) was used for comparisons among multiple groups, and Student’s *t*-test was used for comparisons between two groups. A *p*-value of less than 0.05 (* *p* < 0.05) was considered statistically significant.

## 3. Results

### 3.1. Inhibitory Effect of Baicalin on Infectious Bronchitis Virus Replication in CEK Cells

To investigate the impact of baicalin on Infectious Bronchitis Virus (IBV) replication, different treatment schedules were designed: cells pre-treated with baicalin (20 µg/mL) for 2 h before IBV infection (Baicalin + 2 h → IBV), cells infected with IBV followed by baicalin (20 µg/mL) addition at 2 h post-infection (IBV + 2 h → Baicalin), and cells infected with a mixture of baicalin (20 µg/mL) and IBV co-incubated for 2 h (Baicalin + IBV co-incubation). Controls included cells treated with ribavirin (RT, 10 µg/mL) at 2 hpi (IBV + 2 h → RT) and virus-infected-only group. The mRNA expression level of the IBV nucleocapsid protein gene (N gene) and viral titers were measured at 24 and 48 h post-infection (hpi).

**Figure 1:**
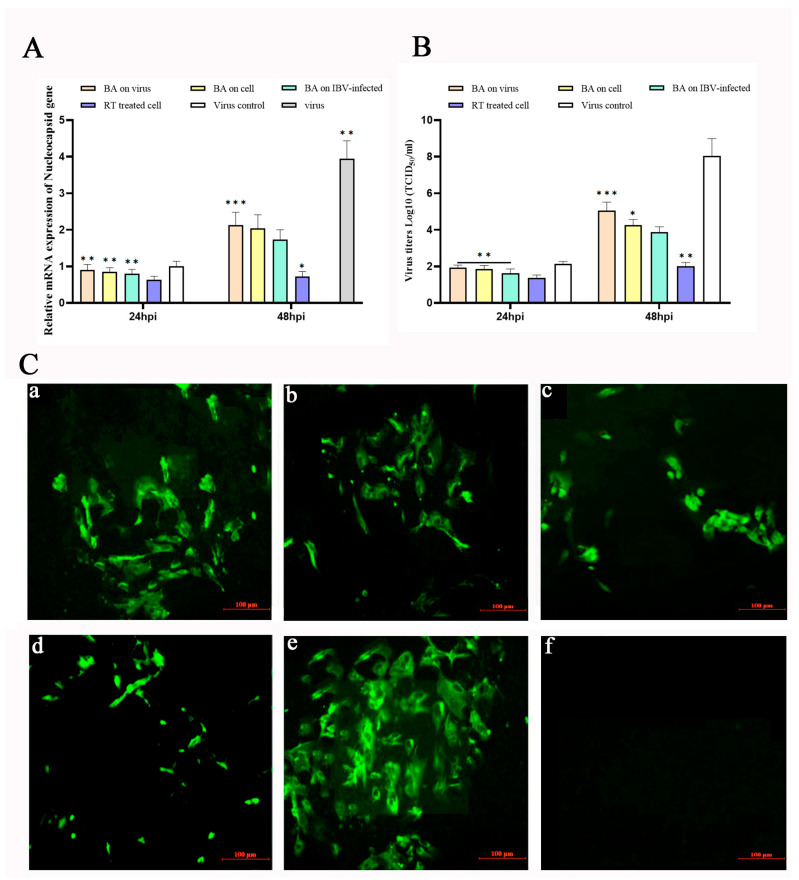
Baicalin inhibits infectious bronchitis virus (IBV) replication in Chicken Embryonic Kidney (CEK) cells. The figure demonstrates the inhibitory effect of baicalin on IBV replication under different treatment schedules. (**A**) Viral N gene mRNA levels were quantified by qRT-PCR at 48 h post-infection (hpi). Baicalin significantly reduced IBV N gene transcription. (**B**) Viral titers were determined by titration assay, confirming the effective suppression of infectious virus production by baicalin treatment. (**C**) Representative immunofluorescence images (48 hpi) visualizing antiviral efficacy under different treatment strategies: (**a**) virus-drug co-incubation; (**b**) pre-treatment with baicalin prior to infection; (**c**) post-infection treatment with baicalin; (**d**) positive control drug (Ribavirin, RT); (**e**) virus-infected control; (**f**) mock-infected control. Scale bar, 100 µm. Data are presented as mean ± SD. * *p* < 0.05, ** *p* < 0.01, *** *p* < 0.001.

At 24 hpi, none of the baicalin (20 µg/mL) treatment groups showed significant suppression of IBV N gene expression, whereas the IBV + 2 h → RT group exhibited a notable inhibitory effect (Figure 1A). In contrast, at 48 hpi, all baicalin-treated groups significantly reduced IBV N gene expression (*p* < 0.05) and suppressed the increase in viral titers (*p* < 0.05). Among these, the IBV + 2 h → Baicalin group demonstrated the most potent antiviral activity, with an inhibition level comparable to that of the IBV + 2 h → RT group, indicating that baicalin effectively suppresses IBV replication in CEK cells (Figure 1B).

To further confirm the inhibitory effect of baicalin on IBV replication, viral protein expression was assessed by indirect immunofluorescence assay (IFA) at 48 hpi. The IFA results corroborated that the IBV + 2 h → Baicalin group exerted the strongest suppression of IBV, consistent with the gene expression and viral titer data, suggesting that baicalin plays a key antiviral role during the early stages of IBV infection (Figure 1C).

### 3.2. Baicalin Temporally Modulates the Transcription of Interferon and Pro-Inflammatory Genes in Response to IBV Infection

To elucidate the molecular mechanism by which baicalin inhibits infectious bronchitis virus (IBV) replication, we further investigated its regulatory effects on key factors of the interferon pathway and the NF-κB-mediated pro-inflammatory pathway, based on the previously confirmed antiviral activity. The following experimental groups were established in chicken embryonic kidney (CEK) cells: baicalin (20 µg/mL) alone, IBV infection alone, pre-treatment with baicalin for 2 h followed by IBV infection, and post-infection treatment with baicalin at 2 hpi, with untreated cells serving as the control. The transcriptional levels of interferon pathway-related genes (MDA5, MAVS, IRF3, IRF7, STAT1, STAT2, MX1, IFN-α, IFN-β) and pro-inflammatory pathway-related genes (IL-1β, IL-6, TNF-α, TLR7) were analyzed by quantitative real-time PCR at 24 and 48 h.

**Figure 2:**
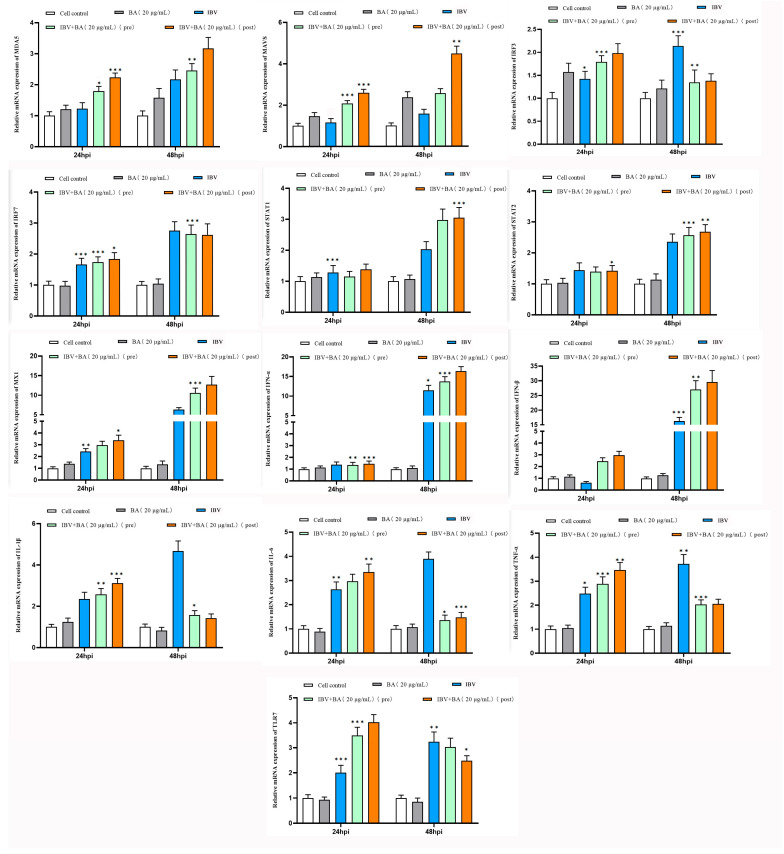
Baicalin modulates innate immune transcription in a treatment- and time-dependent manner. We analyze mRNA expression of interferon (MDA5, MAVS, IRF3, IRF7, STAT1/2, MX1, IFN-α/β) and pro-inflammatory (IL-1β, IL-6, TNF-α, TLR7) pathway components by qRT-PCR in CEK cells under different baicalin treatments at 24 and 48 h. Data are mean ± SD. * *p* < 0.05, ** *p* < 0.01, *** *p* < 0.001.

The results showed that treatment with baicalin alone did not significantly alter the expression levels of any detected genes. At 24 h post-IBV infection, compared with the control group, the expression of IRF3, IRF7, STAT1, STAT2, MX1, IL-1β, IL-6, TNF-α, and TLR7 was upregulated, while IFN-β expression decreased. No significant changes were observed in MDA5, MAVS, or IFN-α, suggesting that IBV infection activates certain innate immune and inflammatory responses but may simultaneously antagonize type I interferon production (Figure 2).

By 48 hpi, baicalin treatment exhibited notable time-dependence and treatment sequence-specific effects. Particularly in the group treated with baicalin after IBV infection, the expression of MDA5, MAVS, STAT1, MX1, and IFN-β in the interferon pathway was significantly and consistently upregulated. In contrast, the expression of IRF7, STAT2, and IFN-α showed no significant fluctuation, while the transcript levels of IRF3 and the pro-inflammatory factors IL-1β, IL-6, TNF-α, and TLR7 were significantly downregulated. Comparatively, pre-treatment with baicalin before viral infection had a weaker modulatory effect on gene expression (Figure 2).

This study demonstrates that baicalin differentially regulates the interferon pathway and the NF-κB-mediated inflammatory response in IBV-infected CEK cells. Baicalin significantly enhanced the expression of the MDA5–MAVS–STAT1–MX1–IFN-β signaling axis, suggesting that it may strengthen the cellular antiviral state by augmenting viral sensing and the type I interferon response. Concurrently, baicalin markedly downregulated IRF3 and key pro-inflammatory factors (IL-1β, IL-6, TNF-α) as well as TLR7, indicating its potential to alleviate IBV-induced inflammatory damage. The more pronounced effect observed when baicalin was administered after viral infection suggests its suitability as a post-infection intervention strategy, effectively suppressing IBV replication through coordinated modulation of the interferon pathway and inhibition of the inflammatory response.

In summary, baicalin remodels the host cell antiviral immune response at the transcriptional level by coordinately regulating the interferon pathway and the NF-κB-mediated inflammatory response, which likely constitutes a key molecular mechanism underlying its inhibition of IBV replication.

### 3.3. Baicalin Induces Biphasic Regulation of IRF3 and IκBα/p65 Phosphorylation at the Protein Level

To further elucidate the molecular mechanism by which baicalin differentially regulates the interferon pathway and the NF-κB-mediated inflammatory response in IBV-infected CEK cells, we analyzed the protein expression and phosphorylation status of key signaling molecules by Western blot.

**Figure 3:**
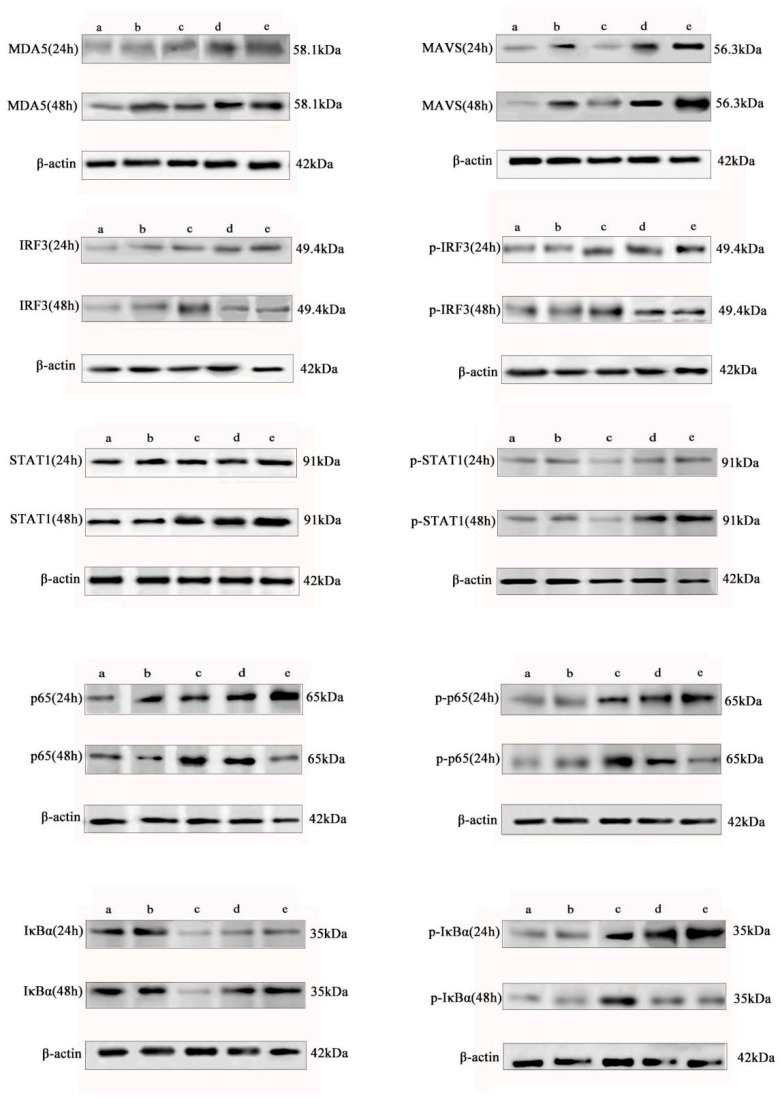
Temporal regulation of innate immune signaling pathways by baicalin at the protein level. Protein expression and phosphorylation of key signaling molecules in the interferon pathway (viral sensor MDA5, adaptor MAVS, and STAT1) as well as major components linking interferon and NF-κB pathways (IRF3, p65, and IκBα) were analyzed by Western blot in CEK cells across different treatment schedules at 24 and 48 h post-infection. The experimental groups included: (a) Mock group, (b) BA (20 µg/mL) group (baicalin only), (c) IBV group (infection only), (d) Baicalin + 2 h → IBV group (Pre-treatment), and (e) IBV + 2 h → Baicalin group (Post-infection treatment). Baicalin was found to sustain the activation of the MDA5–MAVS–STAT1 axis, while exerting a biphasic effect on the phosphorylation of IRF3, p65, and IκBα in a time- and treatment-dependent manner.

The results demonstrated that baicalin consistently enhanced the protein expression of MDA5, MAVS, and STAT1 in the interferon pathway over 24 to 48 h and significantly promoted the phosphorylation of STAT1. In contrast, baicalin exerted a time-dependent biphasic regulatory effect on IRF3 and the NF-κB core subunit p65: it promoted their protein expression and phosphorylation at 24 h, but significantly suppressed both at 48 h (Figure 3).

Further analysis of IκBα protein and its phosphorylation status revealed that baicalin suppressed total IκBα expression while promoting its phosphorylation at 24 h, whereas it enhanced IκBα expression and inhibited its phosphorylation at 48 h. This dynamic pattern aligns with the canonical NF-κB signaling cascade—where IκBα degradation (accompanied by phosphorylation) facilitates p65 activation, and its accumulation suppresses p65 activity—thus confirming that baicalin modulates the transcriptional activity of p65 by regulating IκBα stability (Figure 3).

Notably, the regulatory pattern of baicalin on IRF3 differed from its effect on most interferon pathway factors and instead paralleled the changes in the pro-inflammatory protein p65, suggesting a potential non-canonical role for IRF3 in the baicalin-modulated inflammatory response. Concurrently, the temporal regulation of IκBα further substantiated the biphasic modulatory effect of baicalin on the NF-κB pathway, characterized by initial promotion followed by suppression.

In summary, our data at the protein level confirm that baicalin induces an initial enhancement followed by suppression of the NF-κB-mediated inflammatory response through the temporal regulation of the IRF3 and IκBα/p65 signaling axes. This regulatory mode contrasts with its sustained activation of the MDA5–MAVS–STAT1 interferon pathway, highlighting its multi-target and time-dependent immunomodulatory characteristics. The response pattern of IRF3, which converges with that of pro-inflammatory factors upon baicalin treatment, implies its potential role as a nodal point coordinating interferon and inflammatory responses. Collectively, baicalin balances the interferon response and inflammatory reaction during IBV infection by precisely regulating the expression and phosphorylation of key signaling molecules, thereby contributing to its antiviral effect.

### 3.4. Baicalin Exerts a Time-Dependent Biphasic Control over the NF-κB Pathway by Regulating Downstream Inflammatory Effectors

**Figure 4:**
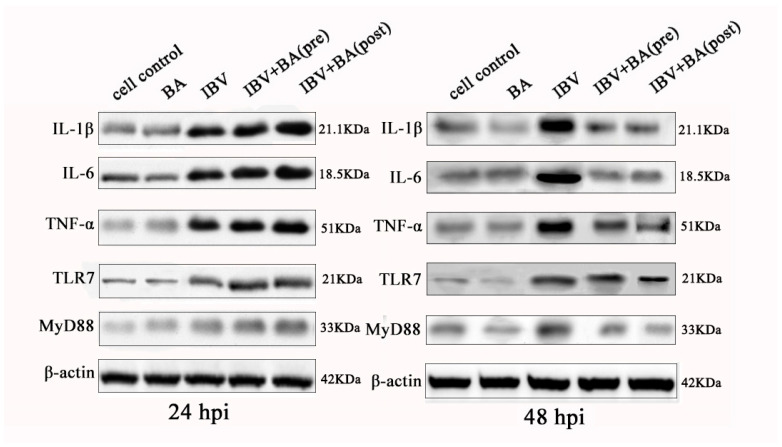
Time-dependent modulation of key NF-κB downstream effector proteins by baicalin. Protein expression levels of the pattern recognition receptor TLR7, its adaptor protein MyD88, and downstream pro-inflammatory effector cytokines (IL-1β, IL-6, TNF-α) were analyzed by Western blot in CEK cells under different baicalin treatment schedules at the indicated time points. Baicalin promoted the expression of these factors at 24 h but suppressed them by 48 h post-infection, demonstrating a biphasic regulatory effect on the NF-κB pathway’s downstream output.

To systematically validate the time-dependent regulatory effect of baicalin on the NF-κB signaling pathway, we further analyzed the protein expression of key downstream factors, including IL-1β, IL-6, TNF-α, TLR7, and its adaptor protein MyD88. Western blot results demonstrated that in IBV-infected CEK cells, baicalin significantly promoted the protein expression of IL-1β, IL-6, TNF-α, TLR7, and MyD88 at 24 h post-infection (hpi), whereas it markedly suppressed the expression of these factors at 48 hpi (Figure 4).

This expression pattern is consistent with the previously observed changes in IκBα/p65 phosphorylation and expression, collectively indicating that baicalin transiently enhances NF-κB pathway activation and promotes the expression of pro-inflammatory factors at the early stage of infection (24 hpi), but subsequently suppresses pathway activity at the later stage (48 hpi), thereby mitigating the inflammatory response.

At the protein level, this study comprehensively reveals that baicalin exerts a clear “promotion then inhibition” time-sequential regulatory effect on the NF-κB pathway and its downstream inflammatory factors. This biphasic modulatory pattern contrasts sharply with its sustained activation of the interferon pathway, highlighting the precise immunomodulatory capacity of baicalin during antiviral responses. Such a time-dependent strategy for inflammation control may help initiate necessary immune defenses at the early stage of infection while avoiding excessive inflammation-induced cellular damage at the later stage, thus providing important mechanistic evidence for baicalin as a candidate anti-IBV agent.

### 3.5. IRF3 Is a Key Mediator of the Anti-Inflammatory and Anti-IBV Effects of Baicalin

To clarify the central role of IRF3 in the baicalin-mediated regulation of the inflammatory response, we knocked down IRF3 expression in CEK cells using siRNA, with a scrambled vector (si-vector) serving as the control. Cells were infected with IBV and subsequently treated with baicalin (20 µg/mL) at 2 h post-infection (hpi). Samples were collected at 48 hpi, and the expression of NF-κB, IL-1β, IL-6, and TNF-α was assessed at both the transcriptional and translational levels by quantitative real-time PCR (qRT-PCR) and Western blot, respectively.

**Figure 5:**
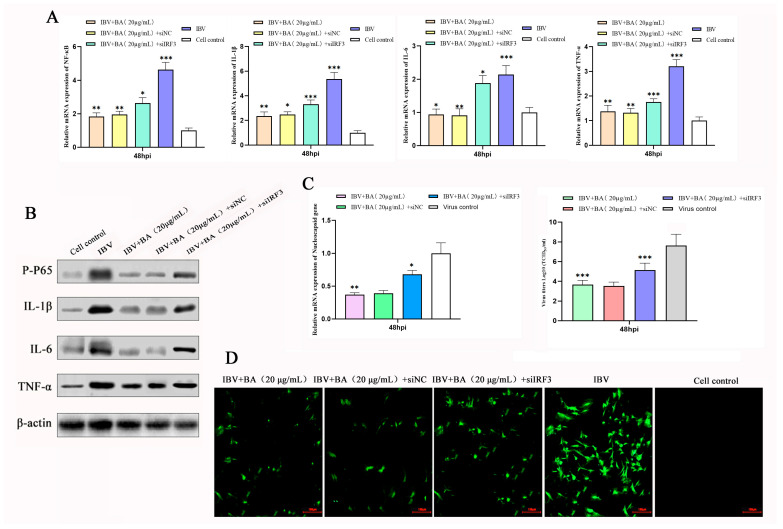
IRF3 is a critical mediator of the antiviral and immunomodulatory functions of baicalin. An IRF3 knockdown model was established in CEK cells to investigate the mechanism of action of baicalin. (**A**) Time-dependent regulation of the transcriptional levels of NF-κB downstream pro-inflammatory cytokines (IL-1β, IL-6, TNF-α) by baicalin following IRF3 knockdown. (**B**) Effect of IRF3 knockdown on the baicalin-mediated suppression of p65 protein phosphorylation and pro-inflammatory cytokine protein expression, as analyzed by Western blot. (**C**) Impact of IRF3 knockdown on the inhibitory effect of baicalin against IBV replication, assessed by viral gene (IBV-N) transcription levels and viral titers. (**D**) The role of IRF3 in baicalin-mediated suppression of IBV replication was visually confirmed by indirect immunofluorescence assay (IFA). Data are presented as mean ± SD. * *p* < 0.05, ** *p* < 0.01, *** *p* < 0.001.

The results showed that IBV infection significantly upregulated the expression of all pro-inflammatory cytokines at 48 hpi, while baicalin treatment effectively suppressed their expression. However, under IRF3 knockdown conditions, the inhibitory effects of baicalin on NF-κB, IL-1β, IL-6, and TNF-α were markedly reversed, as evidenced by a significant recovery in both their mRNA and protein levels (Figure 5A,B). This demonstrates that the loss of IRF3 function effectively compromises the anti-inflammatory effect of baicalin.

To further confirm the role of IRF3 in the antiviral mechanism of baicalin, we simultaneously measured viral replication. The study found that knocking down IRF3 not only reversed the anti-inflammatory effect of baicalin but also severely impaired its ability to suppress IBV replication, leading to a significant rebound in viral titer (Figure 5C,D).

In summary, this loss-of-function study confirms that IRF3 is a critical mediator through which baicalin exerts both its anti-inflammatory and antiviral effects. In the late stage of infection (48 hpi), baicalin downregulates IRF3, thereby inhibiting the NF-κB pathway and the expression of downstream pro-inflammatory cytokines (IL-1β, IL-6, TNF-α), ultimately achieving the dual effects of mitigating the inflammatory response and suppressing viral replication. This finding establishes the central role of IRF3 in the regulatory network of baicalin and provides direct functional evidence for its mechanism of action as a candidate anti-IBV agent.

### 3.6. Baicalin Suppresses the Inflammatory Response by Inhibiting IκBα Phosphorylation and Degradation to Block NF-κB p65 Nuclear Translocation

To further elucidate the molecular mechanism by which baicalin suppresses the NF-κB pathway at the late stage of infection, we investigated whether it acts by affecting IκBα phosphorylation and protein stability, thereby preventing the nuclear translocation of p65—a central step in NF-κB activation. Chicken embryonic kidney (CEK) cells were divided into four groups: blank control, baicalin treatment alone, baicalin combined with the proteasome inhibitor MG132, and MG132 treatment alone. Cells were infected with IBV, treated with baicalin (20 µg/mL) at 2 h post-infection (hpi) for a total of 47 h, followed by co-treatment with MG132 for the final 1 h before sample collection.

**Figure 6:**
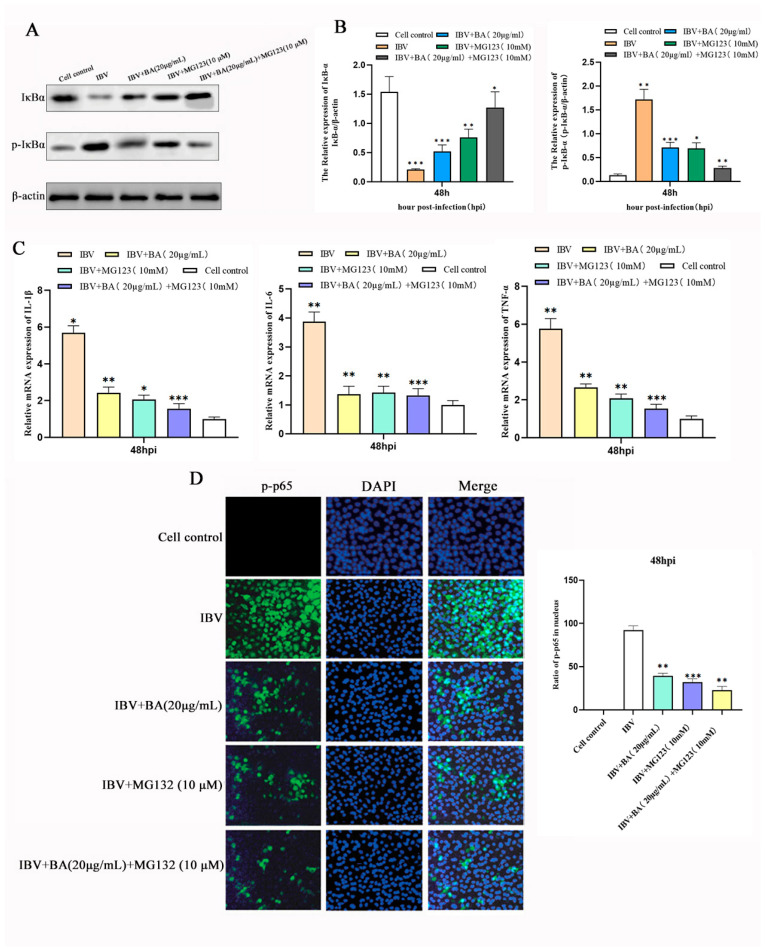
Baicalin suppresses NF-κB pathway activation via a proteasome-independent mechanism. The proteasome inhibitor MG132 was employed to investigate whether baicalin regulates NF-κB by inhibiting IκBα degradation. (**A**) Representative Western blots showing the effect of baicalin on IκBα protein stability in the presence or absence of MG132. (**B**) Quantitative analysis of IκBα protein levels from (**A**). (**C**) qRT-PCR analysis of downstream pro-inflammatory cytokines (IL-6, IL-1β, TNF-α) reveals a synergistic inhibitory effect on their transcription upon co-treatment with baicalin and MG132. (**D**) Immunofluorescence (IFA) analysis confirming that baicalin effectively inhibits the nuclear translocation of phosphorylated p65 (p-p65), an effect that is not dependent on proteasome activity. Data are presented as mean ± SD. * *p* < 0.05, ** *p* < 0.01, *** *p* < 0.001.

Western blot analysis revealed that, compared with the control group, baicalin treatment alone significantly reduced the phosphorylation level of IκBα (p-IκBα) and enhanced its total protein stability (Figure 6A,B). It was further confirmed that the mRNA expression of the NF-κB downstream inflammatory factors IL-6, IL-1β, and TNF-α was also significantly downregulated (Figure 6C). Moreover, baicalin treatment effectively suppressed the phosphorylation of p65 and its translocation into the nucleus (Figure 6D).

In the combination treatment group, MG132 and baicalin exhibited a significant synergistic effect: IκBα protein accumulated substantially, nuclear distribution of p65 was further inhibited, and the transcriptional levels of downstream inflammatory factors were minimized. These results demonstrate that baicalin stabilizes the IκBα protein by inhibiting its phosphorylation and subsequent proteasomal degradation, thereby preventing the activation and nuclear translocation of NF-κB p65.

This study confirms, through multiple aspects—protein stability, subcellular localization, and gene transcription—that baicalin, at the late stage of IBV infection, inhibits IκBα phosphorylation and degradation, enhances IκBα protein stability, and effectively blocks the nuclear translocation and transcriptional activity of NF-κB p65, ultimately downregulating the expression of key inflammatory factors. This mechanism is highly associated with the proteasomal degradation pathway, and the experiment with MG132 further strengthens this conclusion. Thus, we have identified the specific molecular target through which baicalin exerts its “late-phase suppression” in the temporal regulation of the NF-κB inflammatory pathway, providing key evidence for the mechanism underlying its anti-inflammatory effect.

## 4. Discussion

Capitalizing on the unique multi-target antiviral profile of baicalin, this study selected it as a candidate drug against infectious bronchitis virus (IBV). Unlike most chemical agents that act through a single mechanism, baicalin operates synergistically via multiple pathways: it directly targets the virus while simultaneously modulating host cell responses, thereby substantially reducing the risk of antiviral drug resistance [15]. Preliminary investigations confirmed that 20 µg/mL baicalin effectively suppresses IBV replication in vitro and suggested its potential to modulate key cytokines involved in the type I interferon and NF-κB inflammatory pathways, indicating that its antiviral activity is closely linked to innate immune regulation [14].

In this work, we systematically evaluated the time-dependent effects of baicalin on the MDA5- MAVS- STAT1 signaling axis, IFN-β expression, and the NF-κB pathway. The results revealed that baicalin induces sustained upregulation of the MDA5- MAVS- STAT1 pathway and enhances IFN-β production, while exerting a biphasic effect on NF-κB signaling- characterized by initial activation followed by suppression. This temporal pattern of immune regulation underscores a novel mechanism by which baicalin fine-tunes the innate immune response in a time-dependent manner, representing a notable innovation. However, within the innate immune network, MDA5/MAVS serve as upstream sentinels that activate NF-κB, which in turn can amplify the transcription of MDA5 and MAVS, establishing a positive feedback loop to potentiate antiviral signaling [16,17]. The observed sustained upregulation of MDA5/MAVS alongside subsequent NF-κB inhibition thus appears paradoxical from a conventional standpoint. To resolve this discrepancy, we further analyzed the temporal regulation of IRF3. We found that both total and phosphorylated IRF3 followed a trajectory similar to that of NF-κB p65, increasing initially and then declining, whereas IκBα and its phosphorylated form displayed the opposite trend. This coordinated early-phase activation and subsequent attenuation imply that baicalin may initiate a “self-braking” mechanism to achieve precise control over innate immune signaling [18,19].

We propose that this time-dependent regulatory behavior may stem from potent negative feedback loops triggered by early pathway activation. For instance, initial activation of IRF3 and NF-κB could rapidly induce negative regulators such as SOCS family proteins or the deubiquitinase A20, which may subsequently suppress kinase activities including IKK and TBK1 [18]. This would account for the observed re-accumulation of IκBα and the dynamics of its phosphorylation, which trend inversely to p-IRF3 and p-p65. Alternatively, baicalin may directly interfere with the assembly or stability of kinase complexes downstream of MAVS, leading to selective signal attenuation [20]. That said, functional validation of these candidate negative regulators remains incomplete, and the activity profiles of upstream kinases (e.g., TBK1, IKKα/β) as well as potential direct interactions with baicalin require further investigation [21]. Thus, the proposed mechanisms remain speculative at this stage.

It is important to emphasize that all findings presented here are derived from cell-based models. While such systems allow detailed dissection of molecular events, they cannot replicate baicalin’ s pharmacokinetic behavior in vivo or its complex crosstalk with a fully integrated immune system [22]. As a result, we were unable to evaluate the actual protective efficacy of baicalin in infected birds, nor could we assess key innate immune physiological parameters—such as serum lysozyme, complement activity, β-lysins, and phagocytic function [23]—which represent core metrics for evaluating immunomodulatory drug efficacy at the organismal level.

In summary, this study systematically demonstrates that baicalin dynamically modulates the MDA5- MAVS- STAT1 axis and NF-κB/IRF3 signaling, thereby temporally coordinating the innate immune response and likely engaging negative feedback mechanisms to inhibit IBV replication while preventing excessive inflammation. Its multi-target and self-limiting immunomodulatory characteristics make it a promising antiviral candidate with a low risk of resistance. Future work will focus on in vivo validation using IBV-infected chicken models, systematically evaluating baicalin’s impact on key innate immune parameters, and performing cross-level integrative analyses linking molecular pathways to physiological outcomes. These efforts will help fully elucidate its antiviral and immunoregulatory actions across molecular, cellular, and organismal levels, providing a solid foundation for developing baicalin as a green immunomodulatory agent.

## 5. Conclusions

This study systematically elucidates that baicalin effectively suppresses Infectious Bronchitis Virus (IBV) replication in Chicken Embryonic Kidney (CEK) cells through a multi-pathway cooperative mechanism. The principal findings are summarized as follows:

Baicalin demonstrates significant antiviral efficacy against IBV, potently inhibiting viral replication—with a reduction in N gene expression and viral titers upon post-infection administration comparable to ribavirin. Mechanistically, it coordinately modulates innate immune responses by persistently activating the MDA5–MAVS–STAT1 interferon pathway, thereby enhancing IFN-β and MX1 expression and reinforcing the antiviral state. In parallel, baicalin exerts a temporally regulated influence on the NF-κB-mediated inflammatory response: it transiently promotes IκBα/p65 phosphorylation and pro-inflammatory cytokine induction at 24 h, but subsequently suppresses IκBα phosphorylation and degradation, impedes p65 nuclear translocation, and attenuates inflammation by 48 h. Central to this immunomodulatory balance is IRF3, whose knockdown abrogates baicalin’s suppressive effects on NF-κB signaling and inflammatory cytokine production, while concurrently diminishing antiviral activity, indicating its pivotal role as a regulatory node. Further elucidating the anti-inflammatory mechanism, baicalin enhances IκBα stability by blocking its phosphorylation and proteasomal degradation, thereby preventing p65 nuclear translocation. In summary, baicalin combats IBV through synergistic enhancement of the type I interferon response and temporal restriction of NF-κB-driven inflammation, with IRF3 serving as the core coordinator of this immune equilibrium—providing a solid mechanistic basis for its development as an anti-IBV agent.

## Data Availability

The original contributions presented in this study are included in the article/Supplementary Materials. Further inquiries can be directed to the corresponding author.

## Supplementary Materials

The following supporting information can be downloaded at https://www.mdpi.com/article/10.3390/ani15233396/s1; Figure S1: Provide the complete Western blot images for the core genes as supplementary data.

## Appendix A

**Table A1:** Primers of cloning, siRNA and PCR.

Purpose	Name	Sequence (5′ to 3′)
Real-time PCR	IBV-N-F	ATGTCTTCCTCCAAGTCAAGAC
	IBV-N-R	CGACCATGTTCTCCATGATG
Real-time PCR	MDA5-F	TGCTGAAGAAGGAGCTGACC
	MDA5-R	GGTCCTTGTAGTCCTTGGCA
Real-time PCR	MAVS-F	CAGCCAGAAGGAGCTGAACA
	MAVS-R	TCCTGCTGTTCTCCTCCTGT
Real-time PCR	TLR7-F	TTCTGCCTGTGGTCTTCTGG
	TLR7-R	GGCATAGAGGGTGAGGATGG
Real-time PCR	IRF3-F	AAGCAGCCTCAGGTCTACCA
	IRF3-R	GCTGTCCAGGTCCTTGTCAT
Real-time PCR	IRF7-F	CCAGAAGGCTACGAGGACAC
	IRF7-R	GCTCCAGGTCCTTGTCATTG
Real-time PCR	STAT1-F	AAGGACCAGGACTTCACCACA
	STAT1-R	TTCAGGTCGTTCTTCAGGGC
Real-time PCR	STAT2-F	CAAGGAGATCGGCAAGAAGC
	STAT2-R	GGTCCAGGTAGTGGCTTTCC
Real-time PCR	MX1-F	TGCTGACCAAGATGACCAGC
	MX1-R	AGGTCCTGATCCTGGTGA GG
Real-time PCR	IFN-α-F	TGCCTGAAGGAGAAGGTCCT
	IFN-α-R	AGGAGGTGTTGGTGGTGAAG
Real-time PCR	IFN-β-F	AGAAGCAACAGCCAGAGC
	IFN-β-R	TCCTCCTCATTGGCTGTCTC
Real-time PCR	IL-1β-F	AGCTCTACATTCGGCGAAACT
	IL-1β-R	GGTTGGTTGCTGTCTTTCCA
Real-time PCR	IL-6-F	GCTCGCCGGCTTCGAT
	IL-6-R	GGTAGGTCTGAAGGCGAACG
Real-time PCR	TNF-α-F	AGGGACAAGGCTGCCC
	TNF-α-R	GCAAATCGGCTGACGGTGT
Real-time PCR	β-actin-F	TGCGTGACATCAAGGAGAAG
	β-actin-R	TGCTGTAACTTCACCGACCA
Western blot	STAT1-F(Nde I/Xho I)	CCCAAGCTTATGTCCCAGCTT
	STAT1-R	CCGCTCGAGTCAGTCCTTCAGGTC
Western blot	p65-F(Nde I/Xho I)	CCCAAGCTTATGGACGAACTG
	p65-R	CCGCTCGAGCTAGGCAGGCTC
Western blot	IκBα-F(Hind III and Xho I)	CCCAAGCTTATGGCACCTGGC
	IκBα-R	CCGCTCGAGTCAGTCTGCTGC
Western blot	IRF3-F(Hind III and Xho I)	CCCAAGCTTATGGACCGAGAC
	IRF3-R	CCGCTCGAGTCAGTCCTTGTAGTC
Western blot	MAVS-F(Nde I/Xho I)	CCCAAGCTTATGGCTGCCTCC
	MAVS-R	CCGCTCGAGTTAGTCCTTGGTGTC
Western blot	MDA5-F(Nde I/Xho I)	CCCAAGCTTATGGACTCCAGCAGC
	MDA5-R	CCGCTCGAGTCAGTCTTCCTTCTG
Western blot	IL-1β-F(XhoI/EcoRI)	CGCGAATTCATGGCATCAAGGGCTACAAG
	IL-1β-R	CGCCTCGAGTCACACGCAGCGGTTCTT
Western blot	IL-6-F(XhoI/EcoRI)	CGCGAATTCATGCTCTCCCGCCCTCT
	IL-6-R	CGCCTCGAGTCAGGGTCGGGGCATAC
Western blot	TNF-α-F(XhoI/EcoRI)	CGCGAATTCATGAGCACCGAAAGCATGATCC
	TNF-α-R	CGCCTCGAGCTAAGCGACAGCGTGGTTG
Western blot	TLR7-F(XhoI/EcoRI)	CGCGAATTCATGGACCTGAACCCCAACAC
	TLR7-R	CGCCTCGAGCTAGGCCTGGGTGTAGATGAG
Western blot	MyD88-F(XhoI/EcoRI)	CGCGAATTCATGGCAGCGGTTTGGAC
	MyD88-R	CGCCTCGAGTCACAGCTCCAGAGCCTCTTC
siRNA	siIRF3(Sense strand)	r (GAUGAUGAGCUUCUUCAAC) dTdT
	siIRF3(Antisense strand)	r (GUUGAAGAAGCUCAUCAUC) dTdT
