# Supplementary material for: Temporal Modulation of Type I Interferon and NF-κB Signaling by Baicalin Suppresses Infectious Bronchitis Virus Replication and Inflammatory Response
Source: Animals (Basel). 2025 Nov 25;15(23):3396. doi: 10.3390/ani15233396 (PMC12691172; doi:10.3390/ani15233396)
Supplement: Supplementary file 1 [file animals-15-03396-s001.zip › animals-3980267-supplementary.pdf]

Supplementary Materials

Figure S1 Provide the complete Western blot images for the core genes as supplementary data.

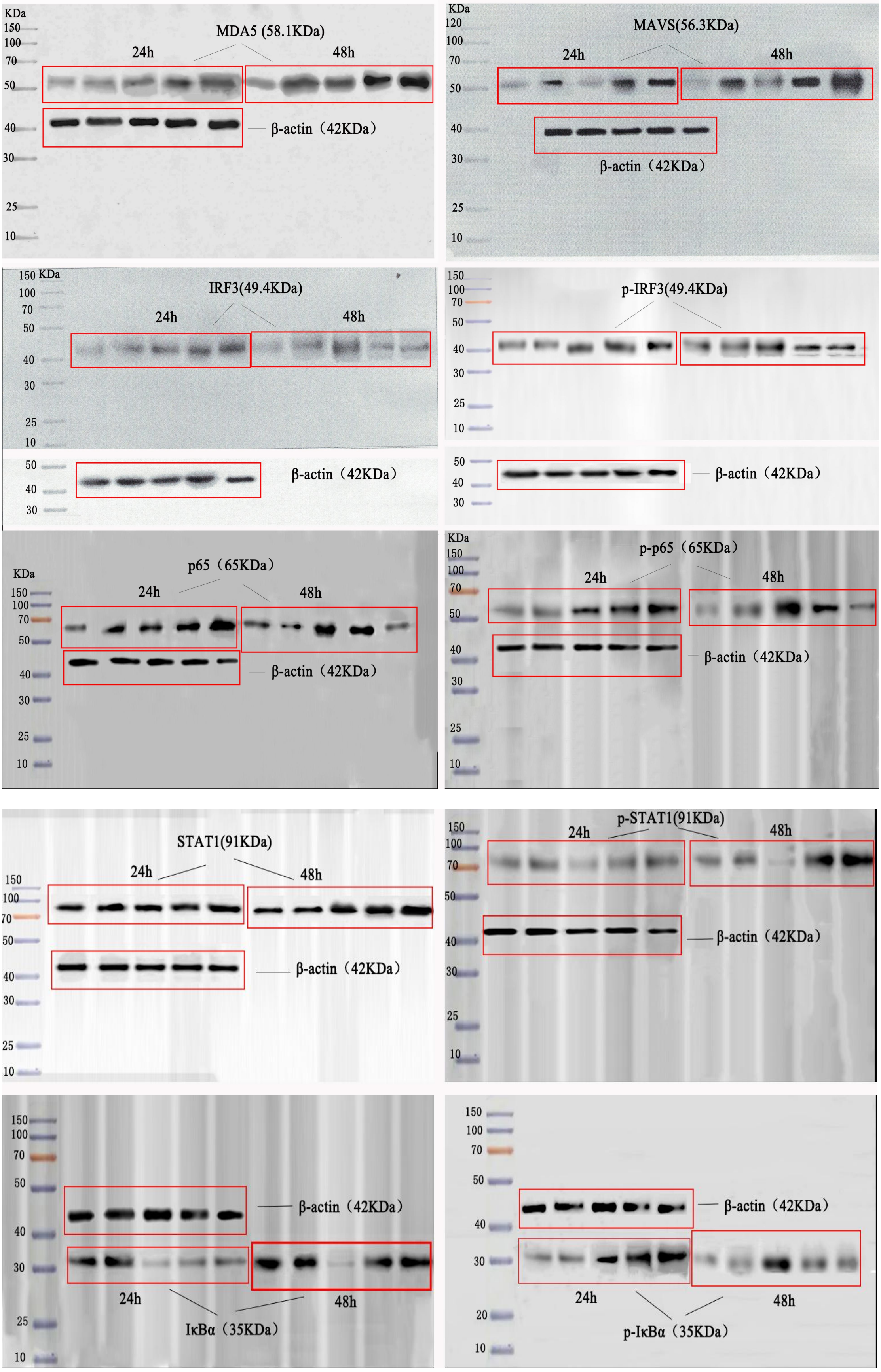

24h

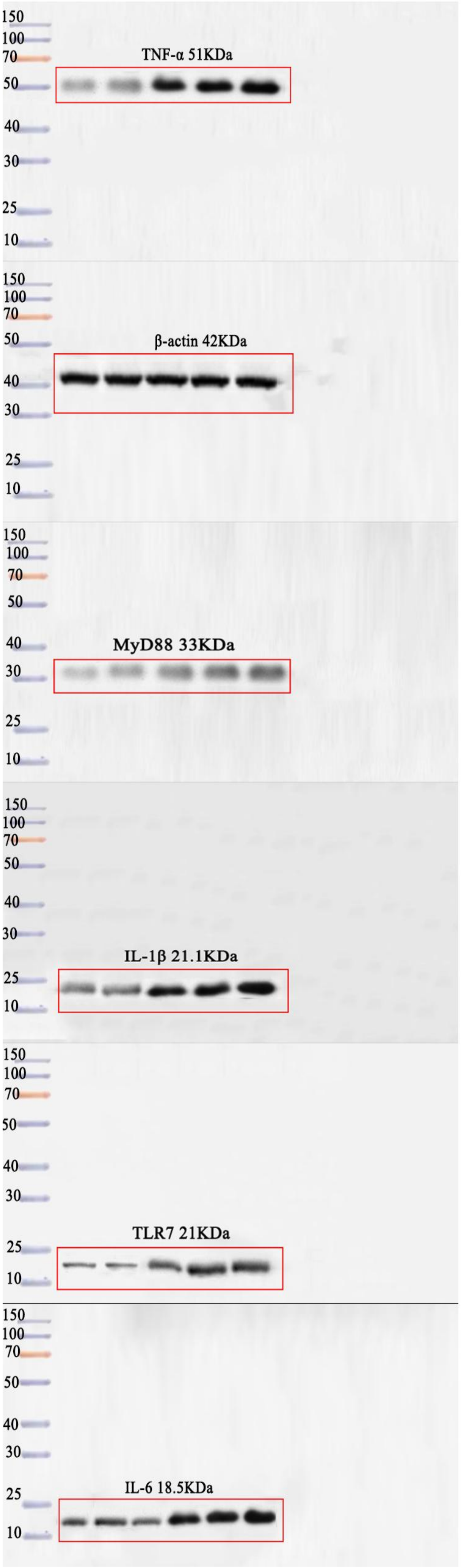
TNF-α 51KDa
β-actin 42KDa
MyD88 33KDa
IL-1β 21.1KDa
TLR7 21KDa
IL-6 18.5KDa

48h

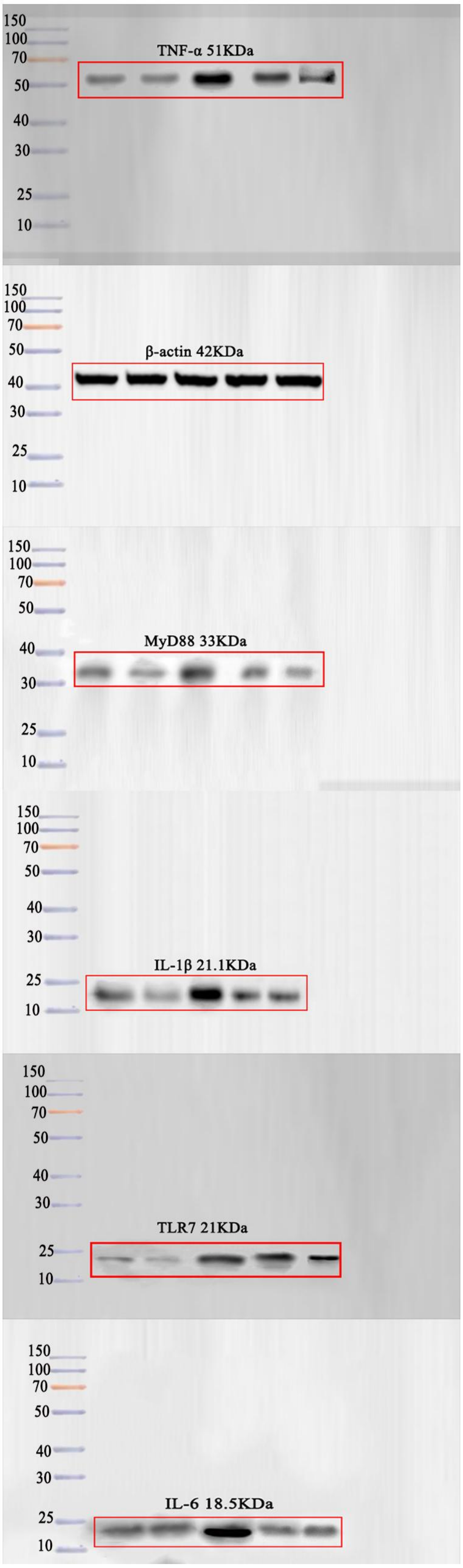
TNF-α 51KDa
β-actin 42KDa
MyD88 33KDa
IL-1β 21.1KDa
TLR7 21KDa
IL-6 18.5KDa
